# Effects of extrusion conditions on the morphological, functional, and sensory properties of soy press cake extrudates

**DOI:** 10.1016/j.heliyon.2024.e32614

**Published:** 2024-06-19

**Authors:** Aditya Bali, Aelita Zabulionė, Shreya Pravin Kumar, Dovilė Liudvinavičiūtė, Laura Pečiulytė, Ramunė Rutkaitė, Per Ertbjerg, Alvija Šalaševičienė

**Affiliations:** a Food Institute, Kaunas University of Technology, Kaunas, Lithuania; b Department of Polymer Chemistry and Technology, Kaunas University of Technology, Kaunas, Lithuania; c Department of Food and Nutrition, University of Helsinki, Helsinki, Finland

**Keywords:** Soy, Texture, Twin-screw extrusion, Press cake, Meat analogue

## Abstract

Abstract: We developed and applied 4 extrusion regimens (moisture content between 30 % and 60 % and temperature from 110 °C to 120 °C) with twin-screw extruder for valorising soy press cakes, byproduct of soy drink (Soy_d_) and tofu (Soy_t_) manufacturing processes, by varying physical conditions of extrusion for improving their morphological, functional, and sensory parameters. The valorised soy press cakes were compared to their respective control samples (Soy_d_ or Soy_t_) both before and after extrusion. Two quantities (3%–6%) of untreated and extruded soy press cakes were utilised to develop meat analogues. Extrusion introduced striations and reduced flakiness on the surface of extruded press cake samples. Press cakes extruded at higher moisture indicated improved water holding and oil holding capacity. Interestingly, the same press cake samples also scored higher for positive indicators (e.g., juiciness) during sensory assessment. Compared with meat analogue control matrix, all meat analogue samples containing varying amounts of extruded press cake exhibited reduced chewiness, with other parameters relatively unchanged. Our results indicate that extrusion of soy press cakes of both Soy_d_ and Soy_t_ origin at 120 °C with 60 % moisture results in improving the morphological, functional, and sensory properties of press cakes, making them suitable for development of meat analogues.

## Highlights

• Different extrusion regimens were developed to apply on soy press cakes.
• Extrusion resulted in an improved morphological and functional profile of soy press cakes.
• Soy tofu manufacturing derived press cakes were overall more favourably perceived by sensory assessors.
• Extrusion at 120 °C with 60 % moisture is recommended for extruding soy press cakes.

## 1 Introduction

The production of plant-based beverages like soy milk generates significant waste material, primarily in the form of press cakes [1]. Plant origin press cakes are commonly utilised as animal feed due to their nutrient profile including significant concentrations of proteins and amino acids, but are not directly utilised as food additive, materials, and ingredients for human consumption. This is primarily due to the presence of antinutrient factors and lack of sensory qualities which may make them distasteful for consumers [2].

Extrusion refers to a food processing technique that involves cooking and shaping plant-based ingredients into various products including puffed snacks and meat-like products. The process takes place in an extruder, a machine that combines heat, pressure, and shear to cook and shape the ingredients [3]. Extrusion cooking is a widely applied processing method for obtaining extrudates of desired properties.

For the production and development of plant-based meat analogue products, high moisture extrusion cooking (HMEC, moisture content up to 70 % w/w water) is mostly utilised as it favourably produces a meat-like fibrous structure in the extrudate after completion of the extrusion cooking process [4]. However, HMEC has some disadvantages. The most significant drawback of products developed using HMEC is the cost of setting up the infrastructure, extra peripherals (cooling die at the end of the barrel) and the cost of producing the goods, which in turn increase the selling price of produced goods [5].

As a result, LMEC (low moisture extrusion cooking) has also been utilised by researchers for the development of extrudates which can be used in the development of plant-based meat analogue products [6,7]. LMEC typically uses moisture levels ranging from 10% to 40 % and temperature ranges between 120 °C and 200 °C [8]. This low moisture level is important for achieving the desired texture and stability of the final product, as well as to prevent spoilage and microorganism growth. Similarly, the temperature range used in LMEC can vary depending on the specific application, ingredients, and desired end-product properties.

The by-product of soy drink and tofu manufacturing processes, known as okara has recently been identified as a potential high protein, high fibre product to be used as animal feed [9,10]. The main difference between okara produced from tofu manufacturing and okara produced from soy drink manufacturing lies in the coagulation step. Tofu production involves intentional coagulation to separate curds from the whey, resulting in a more substantial and moister okara. In contrast, soy drink manufacturing focuses on producing a liquid beverage, so the soy liquid phase is usually filtered, resulting in a drier and finer okara. These factors influence the characteristics and properties of okara from these 2 different sources. Therefore, comparison of okara from different origins (soy drink and tofu manufacturing) is necessary for investigating their potential for development of meat analogue products.

This study aims to apply low and high moisture extrusion regimens on okara sourced from soy drink and tofu manufacturing processes, and compare the differences in morphological, functional, and sensory properties of the different extruded press cakes with non-extruded press cake samples.

## 2 Materials and Methods

### 2.1 Raw materials

**Fig. 1:**
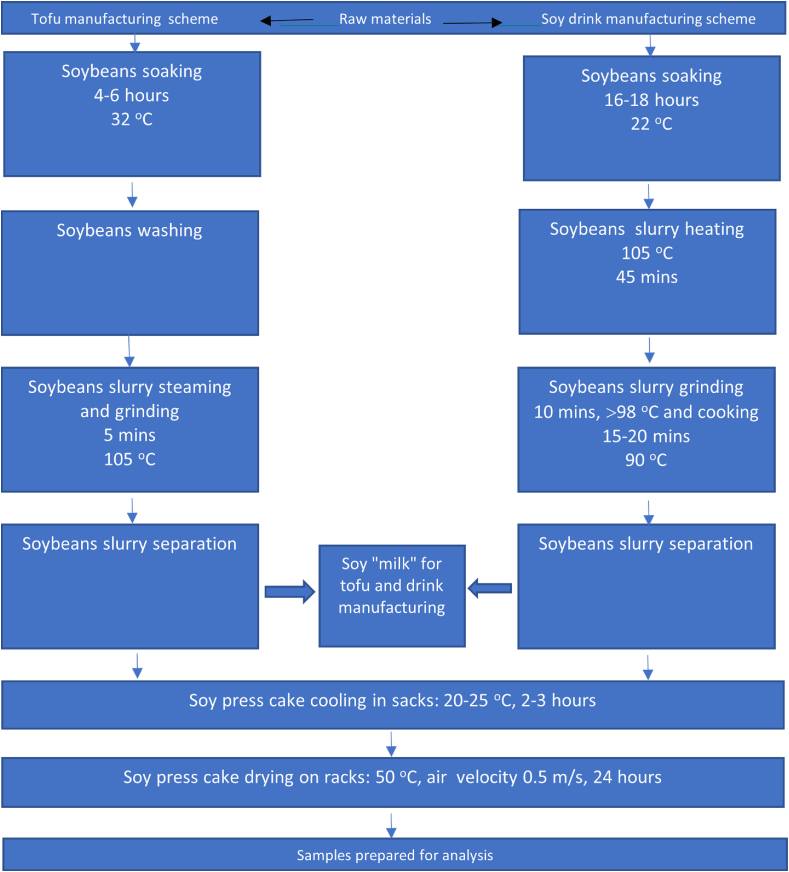
Soy press cakes manufacturing processes.

Two samples of dried soy press cakes, Soy_d_ and Soy_t_ were sourced from Berief Food GmbH, Germany, as residues from soy drink manufacturing and JSC Sojalita, Lithuania, as residues from tofu manufacturing respectively. Dried Soy_d_ samples were provided by the manufacturer, whereas Soy_t_ press cake was dried at 55 °C overnight in a Binder incubator (Binder GmbH). A texturized soy flour for meat analogue matrix (from JSC Imlitex, Lithuania) was used for the study. The technological scheme for obtaining Soy_d_ and Soy_t_ is shown in Fig. 1. For tofu production (Fig. 1), the soybeans were processed in a single tank and the processing time was 45 min. The prepared slurry was boiled at 105 °C with mass being heated by hot water vapor, and the pressure was set to 2 atm. After heating, the slurry was pressed to produce a hot press cake, which was cooled down outside the processing premises by outdoor air. The production of soy drink (Fig. 1) starts with soaking (16–18 h). This is followed by heat treatment, which may last up to 2 h in total. The mass is heated to 100 °C for 45 min followed by grinding in boiling water at a ratio of 1:8 (minimum temperature of 98 °C), finally being boiled at 90 °C for 15–20 min. It is then homogenised, clarified, and cooled. Soy_t_ thermal process takes less than half the time it takes for Soy_d_. Samples were stored at room temperature prior to analysis.

### 2.2 Extrusion process

Pre-treatment: Soy samples (Soy_d_, Soy_t_) were hydrated approximately to 30 % or 60 % moisture before processing. Hydrated samples were allowed to equilibrate overnight at 4 °C.

Extrusion: Pre-treated Soy_d_ and Soy_t_ samples moistened to 30 % or 60 % were extruded using a co-rotating twin-screw extruder ZE25Rx40D-UTXmi (KraussMaffei Berstorff GmbH, Germany) composed of eleven segmented barrels. The screws had a diameter of 26.6 mm, a length to diameter ratio (L/D ratio) of 40:1, and 100 rpm was set as the screw speed. The screws of the extruder consisted of 4 types of conveying elements, which had different size pitches, each type of element was of different length and 2 types of kneading elements of different length. Two kneading elements of the same type were placed in the third barrel segment and one kneading element of different type – in the sixth barrel segment. The full screw configuration is shown in Supplementary Fig. 1. The barrel diameter was 26.9 mm and die head with three circular holes had a diameter of 3.5 mm. Extrusion was carried out under four different conditions for each raw material by varying processing temperature and initial sample moisture. The constant feed rate comprising 30 % of maximal automatic volumetric feeder (calibrated) capacity was set up during the feeding of the samples. Two different temperature profiles throughout the eleven-barrel segments were applied as follows (in °C): i) 18, 65, 85, 95, 100, 105, 110, 110, 110, 110 and product exit temperature (recorded); ii) 18, 65, 85, 95, 105, 110, 120, 120, 120, 120 and product exit temperature (recorded). Extruded samples were marked in Latin numerals with respect to maximum extrusion temperature and moisture of the samples.

I – press cakes extruded at moisture content of 30 % and maximum temperature of 110 °C
II – press cakes extruded at moisture content of 30 % and maximum temperature of 120 °C
III – press cakes extruded at moisture content of 60 % and maximum temperature of 110 °C
IV – press cakes extruded at moisture content of 60 % and maximum temperature of 120 °C

The following process parameters were monitored during extrusion: product temperature in different zones, die pressure and motor load. The throughput was estimated by measuring the mass of the product exiting the extruder per certain period and expressed in kg/h.

The specific mechanical energy (SME) was calculated according to the following expression (Hu et al., 1993):

(1) $$SME\left(Wh{kg}^{-1}\right)=\frac{Screwspeed\left(rpm\right)\times Motorpower\left(W\right)\times Motorload\left(\%\right)}{Maxscrewspeed\left(rpm\right)\times Troughput\left(kg/h\right)\times 100}$$

where extruder constant speed was 100 rpm, max screw speed was 1200 rpm and motor power were set to 26 kW.

### 2.3 Scanning electron microscopy (SEM) analysis

Morphology of extruded and non-extruded soy press cakes was investigated using a FEI Quanta 200 FEG scanning electron microscope. Extruded samples prior to SEM analysis were freeze-dried in a SP Scientific Freeze Dryer (SP Industries, USA). Press cakes were mounted on metal stubs using adhesive tape. Images were taken at a magnification of 5000x.

### 2.4 Preparation of meat analogues

Meat analogues were prepared by addition of 3 % (w/w), and 6 % (w/w) extruded press cake samples to the matrix containing industrially textured soy protein, water, emulsion, and oil. Additional spices were not added to the meat analogue matrix to recognise the base matrix's sensory properties. The emulsion was produced by blending industrial emulsifier, water, and oil. Two batches of each analogue were prepared to carry out analysis of cooked and uncooked meat analogue samples weighing 100 g each. The prepared meat analogues were cooked in a conventional oven with cooking parameters set at 180 °C temperature for 20 min. It was adequate to reach an internal temperature of more than 75 °C. The cooked samples were wrapped using aluminium foil and placed in labelled trays. They were held at a temperature of 60 °C before using them for sensory analysis. The cooked samples were also used for texture and colour analysis.

Samples were coded as per the following abbreviation scheme:

MA-C -: control matrix (from textured soy protein, water, emulsion, and oil) without extruded press cakes
MT -: meat analogue matrix supplemented with extruded Soy_t_ press cakes. Latin numerals from I-IV denote conditions under which press cakes were extruded (see section 2.2 and 3 % or 6 % indicates the amount of press cakes in matrix (w/w)

MD - meat analogue matrix supplemented with extruded Soy_d_ press cakes. Greek number I-IV shows under which conditions press cakes were extruded (see section 2.2 and 3 % or 6 % indicates the amount of press cakes in matrix (w/w).

### 2.5 Analysis of functional properties of extruded and non-extruded press cakes, and meat analogues

#### 2.5.1 Water holding capacity and oil holding capacity

Determination of water holding capacity (WHC) and oil holding capacity (OHC) was performed using the method described by Sadh et al., 2018 [11] with some modifications. Slurries of samples in water were prepared by adding 0.8 g of sample to 15 mL pre-weighted centrifuge tube and pouring 12 mL of distilled water and shaking manually for 1 min. The tubes were centrifuged for 10 min at 3000×*g*. After centrifugation the fugate was discarded and the tubes were weighed again. The WHC or OHC was expressed as grams of water or oil, respectively, bound to per gram of sample.

#### 2.5.2 Cooking loss

The meat analogues of known weight were cooked in a convection oven at 180 °C for 20 min. The samples were allowed to cool to 60 °C and then weighted again. Calculation of cooking loss was done by subtracting the initial weight and the final weight, further dividing it by the original weight of the samples. The cooking loss was expressed as a percentage.

### 2.6 Texture analysis of meat analogues

The texture profile analysis was carried out for samples of cooked meat analogues using Instron 3343 machine (Instron Engineering Group, High Wycombe, England) equipped with a load cell of 1 kN. The samples were cut into cubes measuring 2.0 cm^3^ on each side. The samples were compressed perpendicularly to their largest surface using a cylindrical probe with a diameter of 0.5 cm. The testing conditions were done via two cycles consecutively at a compression of 70 % and crosshead movement at a speed of 1 mm/s.

### 2.7 Sensory analysis of meat analogues

A quantitative descriptive analysis using a 12-point scale was performed by a sensory panel made up of 6 panellists. The panel was tasked with evaluating the meat analogues' sensory characteristics and determining optimal extrusion conditions such as temperature, humidity, the proportion of press cake in the matrix, and optimal storage temperature for the raw meat analogues. A suitable amount of press cake added in the matrix and optimal storage temperature of the raw meat analogues. The scoring range was from 1 to 12, where 1 indicated ‘not intensive’ and 12 - ‘very intensive’.

### 2.8 Statistical analysis

All the experiments were carried out in triplicates. The results were reported as mean ± standard deviation (SD) using Microsoft excel. The one-way analysis of variance (ANOVA for Excel, version 2.2) was used to consider significant differences in the values of extrusion parameters. Duncan's multiple-range test was applied for the calculation of the significant differences among the values of characteristic parameters at probability level p < 0.05. A two-way ANOVA was conducted to assess significant differences in extruded press cake samples, followed by LSD (Least significant difference) post hoc tests. A three-way ANOVA was utilised to evaluate significant differences in meat analogue samples, focusing on the effects of independent factors and their interactions. Results were considered statistically significant at p ≤ 0.05. The analysis was performed using IBM SPSS 27.0 software (IBM Corporation, New York, USA).

## 3 Results & discussion

### 3.1 Extrusion of soy press cakes

In this study, extrusion was conducted on soy press cakes to enhance their suitability for the preparation of meat analogues. Extrusion was applied to soy press cakes with differing moisture and temperature conditions to obtain extrudates with desirable morphological, functional, and sensory properties.

Two parameters (extrusion temperature and moisture) were modulated to produce different samples (see Table 1). Preliminary tests showed that when maximum temperature of the barrel zones (T_max_) was higher than 120 °C, press cakes started to burn. Therefore, two extrusion regimes with lower temperature (T_max_ of 110 °C and 120 °C) were utilised for experiments. The sample output temperature was also monitored at the die and found to be the same as set during the extrusion process, i.e., either 110 °C or 120 °C.

**Table 1:** Parameters of extrusion of Soy_t_ and Soy_d_ press cakes.

Sample	Moisture (%)	*Tmax* (°C)	Motor load (%)	Die pressure (bar)	Throughput (kg/h)	*SME* (Wh kg^−1^)
Soy_t_	30	110	6.0 ± 0.1^c^	26 ± 1^f^	3.4 ± 0.1^a^	38.5 ± 0.4^e^
	30	120	6.3 ± 0.3^c^	25 ± 1^e^	3.1 ± 0.3^a^	44.0 ± 0.5^f^
	60	110	4.0 ± 0.1^a^	6 ± 1^a^	8.5 ± 0.4^b^	10.2 ± 0.7^b^
	60	120	4.0 ± 0.1^a^	7 ± 2^b^	7.5 ± 0.2^b^	11.6 ± 0.6^b^
Soy_d_	30	110	12.0 ± 0.1^e^	21 ± 1^d^	10.7 ± 0.3^f^	24.2 ± 0.6^d^
	30	120	12.0 ± 0.2^e^	19 ± 1^c^	12.7 ± 0.5^c^	20.4 ± 0.7^c^
	60	110	4.7 ± 0.3^b^	6 ± 2^a^	13.1 ± 0.5^cde^	7.7 ± 0.9^a^
	60	120	5.0 ± 0.1^b^	6 ± 2^a^	14.1 ± 0.4^e^	7.7 ± 0.6^a^

The specific mechanical energy (SME) is an important parameter when assessing extrusion process and is defined as the mechanical energy input required to obtain a unit weight of material through the extruder [12]. From data presented in Table 1 it is obvious that the moisture content of press cakes had more influence on SME than the temperature of the barrel. It was observed that the value of SME was lower for both soy press cakes as the humidity of press cake was increased and it is consistent with the data from other studies using twin-screw extruders [12].

Comparing the SME for Soy_t_ samples, it is apparent that SME of Soy_t_ samples was higher at 30 % moisture than at 60 % moisture. A similar trend was observed for Soy_d_ samples, where the SME of Soy_d_ samples extruded at 30 % moisture was higher than samples extruded at 60 % moisture. In general, increasing moisture content of raw soy press cake led to decreased viscosity in the barrel. This reduced the conversion ratio of mechanical energy to heat energy, decreasing the force necessary to push the wet mass through the die [12]. Consequently, motor load and the value of SME decreased, as shown in Table 1. It can also be noticed that increasing moisture of the press cakes lower die pressure and increases throughput of the press cake mass through the extruder. This can be explained by the fact that press cakes at different moisture also have different stickiness and thus are not flowing equally through the barrel. Therefore, higher moisture results in a less viscous press cake mass, which flows more easily. This in turn shortens the mean residence time of the press cake in the extruder barrel and the press cake mass can pass through the die more readily, and these findings are consistent with data presented by Helmick and colleagues [13].

### 3.2 Morphological characteristics of non-extruded and extruded press cakes

#### 3.2.1 Surface morphology

**Fig. 2:**
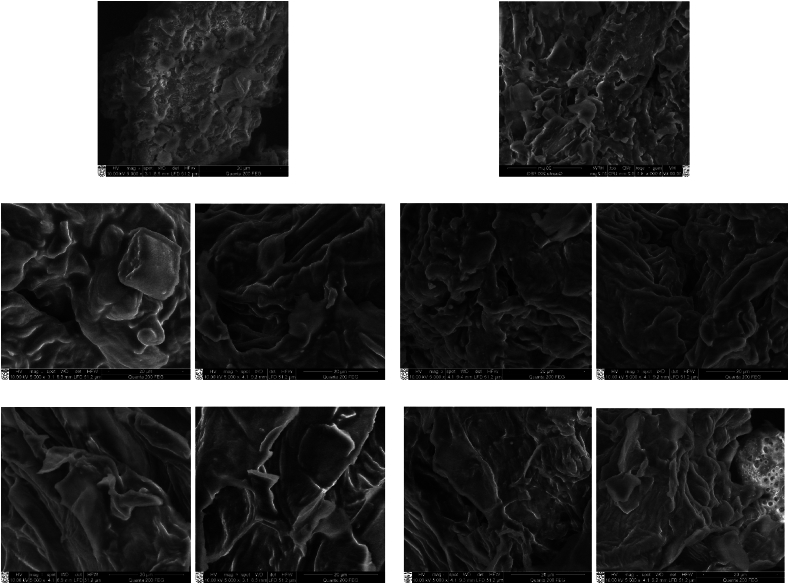
SEM images of non-extruded Soy_t_ (A); Soy_t_ I (1A); Soy_t_ II (2A); Soy_t_ III (3A); Soy_t_ IV (4A); non-extruded Soy_d_ (B); Soy_d_ I (1B); Soy_d_ II (2B); Soy_d_ III (3B); Soy_d_ IV (4B). Magnification of x5000.

Scanning electron microscopy (SEM) was utilised to examine the structural morphology changes of soy press cakes resulting from extrusion under various conditions. SEM images, presented in Fig. 2, illustrate the difference between untreated and extruded soy press cakes samples.

All extruded press cakes were subjected to freeze-drying prior to SEM analysis. It is evident that the structure of both non-extruded soy samples is similar, that is, the surface of the particles is quite flat with visible flaking, though the flaking on the surface of Soy_d_ particles is more apparent (Fig. 2, samples A and B).

Meanwhile, in SEM images of extruded press cakes some morphological changes can be observed at the surface level. After extrusion, independently of the processing temperature or moisture of the samples, strands were visible on the surface of the particles, also some voids appeared. Extruded press cakes at a moisture content of 30 % (samples 1A and 1B) show some plasticity, especially it is visible in extruded Soy_t_ press cakes. As the material cools down, it sets into a porous structure. In present study the formation of porous surfaces are clearly visible for press cakes extruded at higher moisture content (60 %) and temperature (120 °C) as the surface is more strand-like and more holes occur (samples 4A and 4B).

Scanning electron microscopy revealed clear differences in surface morphology between untreated and extruded soy press cake samples. The flakiness and flatness of non-extruded soy press cakes disappeared upon extrusion, and we observe the presence of few threads like striations and some gaps at the surface, indicating that the extruded press-cake is not as flat as it was prior to extrusion. According to Aguilera and colleagues [14] during extrusion at the die “puffing” of the material occurs, which is mainly due to flashing-off of superheated vapor and to the release of normal stresses. This results in the difference in surface topography of the extruded material, causing it to become texturized. The specific mechanical energies of soy press cakes from soy drink and tofu manufacturing pro-cess also support the findings of high-resolution imaging via scanning electron microscopy. Specific mechanical energy has a significant impact on the morphological and textural properties of the extrudate obtained from soy press cake. For example, it has been reported that an increase in SME of extrusion is directly correlated with an increase in the tensile strength of extrudates, and leads to a darker colour [15]. Tensile strength and colour of extrudates are directly related with the protein structure and carbohydrate profile of extrudates, where tensile strength is a textural characteristic and colour is a morphological characteristic. By controlling the specific mechanical energy, it is possible to influence factors such as expansion ratio, bulk density, texture, and nutritional characteristics of the final product. Adjusting the specific mechanical energy can help achieve the desired extrudate characteristics in terms of texture, shape, and size.

#### 3.2.2 Water-holding and oil-holding capacity

In developing food texture, water-holding capacity plays an important role. WHC reflects the protein's ability to retain water and shape the protein gel network. The higher the value of WHC, the greater the juiciness of meat analogue [16]. Similarly, a higher OHC can assist in improved emulsification of meat analog ingredients and may assist in improving the colour of meat analogue products as they are better carriers of carotenoids and related compounds [17]. The WHC and OHC affects the textural properties of press cakes, specifically due to the method used to produce them. Combined with varying extrusion parameters, these are the main factors which contribute to the differences in natural texture of okara produced from either tofu manufacturing, where it is generally softer and moister due to high moisture content, or from soy drink manufacturing where natural consistency is reported to be drier and finer.

Our study showed that with increasing temperature and humidity in the extrusion process, the water-holding capacity of extruded Soy_d_ samples increased compared to non-extruded Soy_d_. For Soy_t_ group of samples, extrusion also increased the water holding capacity of all extruded samples compared to non-extruded Soy_t_ samples, but the increase was not as high as it was for Soy_d_ group of samples (Table 2, Table 3) (see Table 4).

**Table 2:** Water-holding and Oil-holding capacities of Soy_d_ (values presented in g/100g dry matter).

Samples	WHC	OHC
Soy_d_	5.572 ± 0.47^a,A^	1.99 ± 0.23^a,A^
Soy_d_ I	6.095 ± 0.04^b,B^	2.03 ± 0.10^b,B^
Soy_d_ II	6.135 ± 0.17^c,C^	2.48 ± 0.12^c,C^
Soy_d_ III	6.658 ± 0.12^d,D^	2.65 ± 0.18^d,D^
Soy_d_ IV	6.877 ± 0.12^e,E^	2.97 ± 0.11^e,E^

a-e within the same column indicate significant difference for temperature, 2-way ANOVA, p < 0.05, A-E within the same column indicate significance for humidity, 2-way ANOVA, p < 0.05

**Table 3:** Water-holding and Oil-holding capacities of Soy_t_ (values presented in g/100g dry matter).

Samples	WHC	OHC
Soy_t_	4.15 ± 0.01^a,A^	1.71 ± 0.18^a,A^
Soy_t_ I	4.28 ± 0.19^b,B^	1.99 ± 0.25^b,B^
Soy_t_ II	4.57 ± 0.15^c,C^	2.42 ± 0.15^c,C^
Soy_t_ III	4.81 ± 0.10^d,D^	2.62 ± 0.17^d,D^
Soy_t_ IV	5.45 ± 0.17^e,E^	2.93 ± 0.10^e,E^

a-e within the same column indicate significant difference for temperature, 2-way ANOVA, p < 0.05, A-E within the same column indicate significance for humidity, 2-way ANOVA, p < 0.05

The highest water-holding and oil-holding capacity were demonstrated in Soy_d_ press cakes extruded at 120 °C and 60 % humidity. The extruded Soy_d_ samples showed a significant increase in water-holding capacity compared to non-extruded Soy_d_. Non-extruded Soy_t_ also had the lowest water-holding capacity among samples in its group, but the extrusion process was able to improve the water-holding capacity of extruded Soy_t_ samples only by small amounts (Table 3). The water-holding capacity of extruded press cakes increased with increasing temperature and increasing humidity and evolved similarly for both types of soy samples.

During extrusion, a new spatial structure of proteins is likely to form which may result in improved water retention. As okara produced from soy drink manufacturing processes is usually drier and finer compared to okara produced from tofu manufacturing process, we observe an increase in WHC in Soy_d_ press cakes before and after extrusion which may be due to protein unfolding upon extrusion cooking. Increased WHC can create an analogue matrix which can hold more moisture and would lead to a more pleasant and acceptable in-mouth feel. Our results are in tandem with the study conducted by Wi and colleagues [18] who reported increasing water-holding capacities which was demonstrated by the exposure of hydrophobic groups and the unfolding of protein structure with increasing temperature after extrusion.

Oil holding capacity of extruded soy press cakes also increased compared to non-extruded press cakes. OHC for non-extruded press cakes were observed for Soy_d_ at 1.99 ± 0.23 g/100g dry matter and Soy_t_, at 1.71 ± 0.18 g/100g dry matter (Table 2). After extrusion, the oil holding capacity increased to a maximum of 2.93 ± 0.1 g/100g dry matter for Soy_t_ (comparing non-extruded press cake to highest result, which was conceived by extrusion at 120 °C and 60 % humidity, Table 3) and to a maximum of 2.97 ± 0.11 g/100g dry matter for Soy_d_ (at same comparison). This increase occurred as the temperature and humidity of the extrusion increased. This was reported due to enhanced porosity of fibres present in press cake samples, which gave rise to the ability to trap oil droplets. A high lipid retention capacity is necessary not only to create a juicy texture, but to also prevent the creation of fat pockets, which apart from being unappealing to the user can also lead to the degradation of products [18].

Holding of fat with other biomolecules, in particular proteins and carbohydrates affects the texture properties and other qualities of food. In formulations, the ability of proteins to absorb and retain fat and to interact with lipids in emulsions and other food systems is important. Oil-holding in meat analogue matrix occurs due to the interactions between the proteins and fats in the product. When press cake is processed and combined with other ingredients to create a meat analogue, the proteins in the press cake can form a network that entraps and holds the oil within the product. This network helps to prevent the oil from leaching out of the product, giving the meat analogue a more moist and juicy texture [18]. Moreover, the hydrophobic domains of proteins influence fat absorption. Therefore, depending on the extent of the polar side chains of amino acids present on the surface of protein molecules, the oil-holding ability of the samples can differ. When compared with the study conducted by Wu and colleagues where the press cakes were fermented, our results from WHC and OHC analysis are quite similar and lie within the same range.

### 3.3 Analysis of meat analogues produced from non-extruded and extruded press cakes

#### 3.3.1 Texture profile analysis of prepared meat analogues from extruded and non-extruded press cake samples

Texture profile analysis (TPA) is useful as the textural characteristics derived from TPA correlates with the sensory assessment of textural characteristics [19]. The capability to retain fat, oil, and water-holding capacity, as well as their gelling and emulsifying properties which can be measured via texture analysis are all factors to consider. The sensory characteristics and rheological properties of different foods have been compared using parameters found in texture profile analysis such as adhesiveness, cohesiveness, springiness, and gumminess [20]. The results of texture profile analysis of press cake samples have been displayed in Table 3a. Samples are coded with following abbreviations.

• MA-C – meat analogue control, without press cakes.
• MT – meat analogue with press cakes from tofu production
• MD – meat analogue with press cakes from soy drink production.
• I-IV – shows which extrusion regime was used for certain press cake that was used in meat analogue (as mentioned in methodology section 2.2)
• 3 %, 6 % = amount of extruded press cakes added to meat analogue matrix.

The texture profile analysis depicts that the addition of extruded press cakes to the meat analogue matrix did show some significant effect and improved its springiness and gumminess as compared to the control meat analogue. The results of texture profile analysis show similar characteristics of meat analogues with extruded press cakes to that of meat patties. Springiness of meat analogue samples containing extruded Soy_t_ and Soy_d_ press cakes had values between 1.5– 5.8 mm and 2.4–2.9 mm, respectively. Applied extrusion regimens had a significant effect on the springiness of samples, except for sample MT I 3 % (p < 0.05). Gumminess lowered for all samples with the addition of extruded press cakes to meat analogue matrix. All values were statistically significant (p < 0.05).

#### 3.3.2 Sensory analysis of prepared meat analogues from extruded and non-extruded press cake samples

Sensory assessment by trained panellists were conducted on the same samples as in subsection 3.3.1. The results of sensory assessment of the prepared meat analogues are shown in Tables 4 and 5.

**Table 4:**
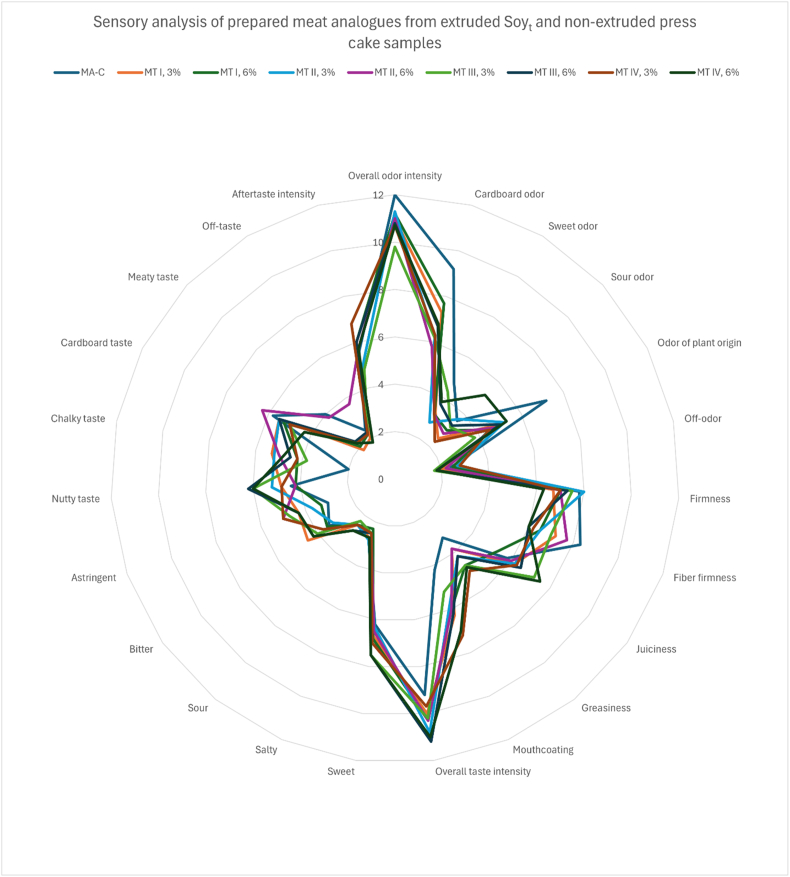
Sensory analysis of prepared meat analogues from extruded Soy_t_ and non-extruded press cake samples.

**Table 5:**
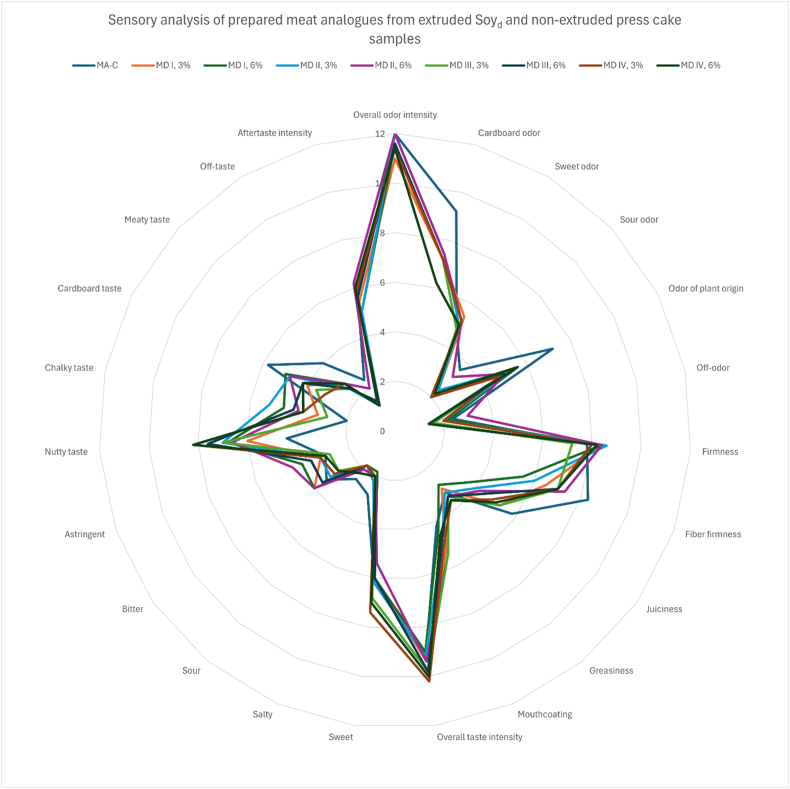
Sensory analysis of prepared meat analogues from extruded Soy_d_ and non-extruded press cake samples.

Sensory evaluation of the samples was comparable between pure meat analogue sample and meat analogue samples prepared from either 3 % or 6 % of Soy_d_ or Soy_t_ press cake (Tables 4 and 5). For example, almost all samples (including control sample) rated extremely high on overall odour intensity (around 11/12 out of 12). Differences were most clearly observed in nutty taste profile, where control samples were rated almost half of the score (4.4/12) that was assigned to samples produced by the addition of Soy_d_ press cakes (between 7 and 8/12). For meat analogues prepared with the addition of Soy_t_ press cake, the nutty taste profile was not markedly different from control samples.

Sensory analysis revealed that the most test samples, including the control, received low ratings for negative sensory descriptors such as off odour, bitter taste, sour taste, and off taste. Interestingly, all samples including control also reported lower scores for the sensory descriptor “meaty taste”. Instead, a pronounced nutty taste was reported to be more prevalent across all samples prepared with Soy_d_ and Soy_t_ press cakes.

Sensory analysis revealed Soy_d_ press cake, extruded at 120 °C with 60 % moisture added to meat analogue samples in 6 % concentration scored the highest in overall consumer acceptance which is aggregated from multiple criteria among panellists. This is an interesting result as in natural state, okara produced from soy drink manufacturing processes is reported to be finer and drier compared to okara produced via tofu production. The product's appearance is crucial for attracting consumers and establishing expectations, plays a significant role prior to consumption. As a result, it is necessary to provide good sensory qualities that can be experienced before and after consumption of the product [21] To create positive expectations, meat analogues should have a similar overall appearance to familiar meat products. The recreation of the distinctive appearance, taste, flavour, aroma, texture, mouthfeel, and moistness of conventional meat products is another challenge for developing meat analogues [22].

## 4 Conclusion

This study rigorously investigated the impact of extrusion conditions on the morphological, functional, and sensory attributes of soy press cake extrudates in the context of meat analogue development.

Morphological analysis revealed that extrusion, particularly under higher moisture regimens, induced notable porosity on the surface of all extrudates, both from soy drink and tofu manufactured press cakes. Additionally, a marked increase in plasticity was observed in extrudates with lower moisture content. Notably, surface flakiness diminished across all extruded samples, accompanied by the formation of distinct striations.

Functional properties of press cakes significantly improved following extrusion. Maximum water-holding capacity was observed in press cakes extruded at 120 °C with 60 % alongside enhanced oil-holding capacity.

Texture analysis revealed that chewiness diminished in all samples with extruded press cakes, whereas springiness was elevated in two specific meat analogue samples supplemented with 3 % soy tofu manufacturing-derived press cake. In other samples, springiness was lower compared to the control.

Sensory evaluation results demonstrated that the meat analogues enriched with 6 % extruded press cakes from both tofu and drink manufacturing processes under conditions of 60 % humidity and 120 °C achieved the highest acceptability scores for most of the positive sensory attributes and minimal scores for negative descriptors.

In summary, this study elucidates that varying extrusion regimens distinctly influence the morphological, functional, and sensory properties of the resultant extrudates. The origin of the soy press cake (soy drink or tofu manufacturing) also pays a critical role, as evidenced by the sensory assessment. While soy drink manufacturing-derived press cakes manifested a heightened “meaty” taste, soy tofu manufacturing-derived press cakes were overall more favourably perceived. It is however important to note that soy drink press cakes and soy tofu press cakes utilised in this study were not necessarily obtained from the same source, which may impact the findings of this study.

Thus, our findings endorse the use of soy press cake from both soy drink and tofu manufacturing, extruded at 120 °C with 60 % moisture in a twin-screw extruder, for meat analogue product development. However, it is crucial to recognise that the extrusion conditions substantially dictate the final product's desired characteristics.

## Ethics and Consent statement

The sensory analysis study was carried out in Lithuania. Ethics Committee approval was not required for this study for the following reasons. According to the institution's (nationally applicable) research ethics requirements, ethics committee approval is required if: the research involves the use of a human embryo, human body cells or tissues; includes of socially vulnerable persons; use clinical or interventional methods; use animals in the research; involve tracking and observing humans under natural, non-experimental conditions when individuals are not informed about the research being performed; intended human research involve sensitive topics that may cause psychological harm, confidential personal data (e.g., related to ethnic origin, religious, philosophical, political and other beliefs, health status) be collected and stored during the planned research; the disclosure of which could damage the reputation of the participants, their relatives or other people. None of these circumstances were applicable during sensory evaluation. Participants-evaluators took part in the study of their own free will, were aware of the exact composition of the product to be evaluated, the warnings about possible allergens, the purpose of the study and the role of the evaluator in it. Only safe and commercially available ingredients were used in the product under development and no ingredients classified as novel food were used. Although the used press cakes are not routinely sold, they are identical in nature and composition to the raw material from which they are derived. Their chemical and microbiological safety has been ensured and confirmed by laboratory tests. Neither the product itself nor the method of evaluation could have caused any harm to the physical or mental health of the evaluators.

As the information sheet provided to the participants - evaluators contains sensitive information (data identifying the participant-evaluator and the contact details of the participant-evaluator), these documents are not attached to the publication in order not to breach the Data Protection Regulation. In case of an urgent need to see the relevant documents, two options are available.

1. Provide a sample of the unsigned questionnaire to assess the validity of the consent;
2. To disclose the questionnaires only to interested persons who undertake in writing to guarantee the confidentiality of the data received.

Even though agreement to participate in research includes participant - evaluators identity and contacts, evaluation itself was anonymous and results were not assigned to specific evaluator.

## Data availability statement

The data that support the findings of this study are available from the corresponding author upon reasonable request.

## CRediT authorship contribution statement

**Aditya Bali:** Writing – review & editing, Writing – original draft, Conceptualization. **Aelita Zabulionė:** Writing – original draft, Visualization, Validation, Methodology, Investigation, Formal analysis. **Shreya Pravin Kumar:** Writing – original draft, Investigation, Formal analysis. **Dovilė Liudvinavičiūtė:** Writing – review & editing, Writing – original draft, Methodology, Investigation, Formal analysis. **Laura Pečiulytė:** Methodology, Investigation, Formal analysis. **Ramunė Rutkaitė:** Writing – review & editing, Writing – original draft, Methodology, Investigation. **Per Ertbjerg:** Methodology. **Alvija Šalaševičienė:** Writing – review & editing, Supervision, Resources, Investigation, Funding acquisition, Conceptualization.

## Declaration of Competing Interest

The authors declare that they have no known competing financial interests or personal relationships that could have appeared to influence the work reported in this paper.

## References

[1] Trakselyte-Rupsiene K., Juodeikiene G., Alzbergaite G., Zadeike D., Bartkiene E., Ozogul F., Rueller L., Robert J., F, Rocha J.M. (2022). Bio-refinery of plant drinks press cake permeate using ultrafiltration and lactobacillus fermentation into antimicrobials and its effect on the growth of wheatgrass in vivo. Food Biosci..

[2] Ancuta P., Sonia A. (2020). Oil press-cakes and meals valorisation through circular economy approaches. A review Applied Sciences.

[3] Ruiz-Gutiérrez M.G., Sánchez-Madrigal M.Á., Quintero-Ramos A. (2018).

[4] Schmid E.M., Farahnaky A., Adhikari B., Torley P.J. (2022). High moisture extrusion cooking of meat analogs: a review of mechanisms of protein texturization. Compr. Rev. Food Sci. Food Saf..

[5] Morrison Oliver (2022). Why high moisture extrusion could solve alt meat's nutritional as well as structural challenges. Food Navigator.

[6] Hu L., Hsieh F., Huff H.E. (1993). Corn meal extrusion with emulsifier and soybean fiber. LWTâ“Food Sci. Technol..

[7] Jung-Soo L., Hyewon O., Choi I., Suk-Yoon C., Jaejoon H. (2022). Physico-chemical characteristics of rice protein-based novel textured vegetable proteins as meat analogues produced by low-moisture extrusion cooking technology. Lebensm. Wiss. Technol..

[8] Beck S.M., Knoerzer K., Foerster M., Mayo S., Philipp C., Arcot J. (2018). Low moisture extrusion of pea protein and pea fibre fortified rice starch blends. J. Food Eng..

[9] Harthan L.B., Cherney J.R.B. (2017). Okara as a protein supplement affects feed intake and milk composition of ewes and growth performance of lambs. Animal Nutrition.

[10] Aschemann-Witzel J., Gantriis R.F., Fraga P., Perez-Cueto F.J.A. (2021). Plant-based food and protein trend from a business perspective: markets, consumers, and the challenges and opportunities in the future. Crit. Rev. Food Sci. Nutr..

[11] Sadh P.K., Chawla P., Duhan J.S. (2018). Fermentation approach on phenolic, antioxidants and functional properties of peanut press cake. Food Biosci..

[12] Karunanithy C., Muthukumarappan K., Gibbons W.R. (2012). Effect of extruder screw speed, temperature, and enzyme levels on sugar recovery from different biomasses. ISRN Biotechnol.

[13] Helmick H., Tonner T., Hauersperger D., Okos M., Kokini J.L. (2023). Comparison of the specific mechanical energy, specific thermal energy, and functional properties of cold and hot extruded pea protein isolate. Food Res. Int..

[14] Aguilera J.M., Kosikowski F.V. (1976). Ultrastructural changes occurring during thermoplastic extrusion of soybean grits. J. Food Sci..

[15] Fang Yanqiang, Zhang Bo, Wei Yimin (2014). Effects of the specific mechanical energy on the physicochemical properties of texturized soy protein during high-moisture extrusion cooking. J. Food Eng..

[16] Yongfeng A., Cichy K.A., Harte J.B., Kelly J.D., Ng P.K.W. (2016). Effects of extrusion cooking on the chemical composition and functional properties of dry common bean powders. Food Chem..

[17] Kumar P., Sharma N., Ahmed M.A., Verma A.K., Umaraw P., Mehta N., Abubakar A.A., Hayat M.N., Kaka U., Lee S. (2022). J technological interventions in improving the functionality of proteins during processing of meat analogs. Front. Nutr..

[18] Wi G Bae, J, Kim H., Cho Y., Choi M.J. (2020). Evaluation of the physicochemical and structural properties and the sensory characteristics of meat analogues prepared with various non-animal based liquid additives. Foods.

[19] Schreuders F.K.G., Schlangen M., Kyriakopoulou K., Boom R.M. (2021). van der Goot, Texture methods for evaluating meat and meat analogue structures: A review. Food Control.

[20] Nishinari K., Kohyama K., Kumagai H., Funami T., Bourne M.C. (2013). Parameters of texture profile analysis. Food Sci. Technol. Res..

[21] Delwiche J. (2004). The impact of perceptual interactions on perceived flavour. Food Qual. Prefer..

[22] Fiorentini M., Kinchla A.J., Nolden A.A. (2020). Role of sensory evaluation in consumer acceptance of plant-based meat analogs and meat extenders: a scoping review. Foods.

