# Supplementary material for: Effects of extrusion conditions on the morphological, functional, and sensory properties of soy press cake extrudates
Source: Heliyon. 2024 Jun 19;10(12):e32614. doi: 10.1016/j.heliyon.2024.e32614 (PMC11252654; doi:10.1016/j.heliyon.2024.e32614)
Supplement: Multimedia component 1 [file mmc1.docx]

**SUTIKIMAS DALYVAUTI MOKSLINIAME TYRIME**

KTU Maisto instituto, juslinės analizės laboratorija, rengia mokslinį tyrimą:

***„Augalinės kilmės mėsos analogų, su ekstruduotomis sojos išspaudomis, juslinė analizė“***

**Tyrimo tikslas.** Atlikti augalinės kilmės produkto, praturtinto ekstruduotomis sojų išspaudomis, juslinę aprašomąją kiekybinę analizę.

**Tyrimo dalyviai.** Vertintojai – ekspertai, turintys patirties atliekant aprašomąją juslinę analizę.

**Tyrimo metodas.** Tyrimas bus atliekamas laikantis ISO 11035:1994 (Sensory analysis — Identification and selection of descriptors for establishing a sensory profile by a multidimensional approach) reikalavimų. Tyrimo metu vertintojui bus pateikiami skirtingi mėsos analogų mėginiai ir bus prašoma ragaujant gaminius atsakyti į klausimus. Vertinimui skirti mėginiai ir vertinimo klausimynas bus pateikiamas kiekvienam vertintojui asmeniškai, suteikiant privačią erdvę vertinimui. Vertinimas yra anoniminis ir iš vertinimo rezultatų nebus įmanoma nustatyti, kuris vertintojas pateikė atsakymus. Vertinimo trukmė apie 25 minutes.

**Įspėjimas dėl alergizuojančių medžiagų: produkto sudėtyje yra sojos.**

*Tyrimo dalyvio teisės: tyrimo dalyviui* *garantuojamas anonimiškumas ir gautų duomenų konfidencialumas. Apibendrinti tyrimo rezultatai bus panaudoti tik tyrimo tikslui įgyvendinti. Dalyvis iki tyrimo pradžios gali atsisakyti dalyvauti tyrime, tačiau pateikus vertinimą, jo atsiimti nėra galimybės, kadangi vertinimas yra pilnai anonimiškas ir neįmanoma nustatyti, kuris vertinimas priklauso konkrečiam vertintojui. Vertintojai, jaunesni nei 18 metų amžiaus, norėdami dalyvauti tyrime turi pateikti bent vieno iš tėvų (ar globėjų) pasirašytą sutikimą bei išreikšti savo sutikimą dalyvauti tyrime. Iškilus bet kokiems klausimams tyrimo dalyvis gali kreiptis e. paštu* [*Aelita.Zabulione@ktu.lt*](mailto:Aelita.Zabulione@ktu.lt)*. į KTU Maisto instituto, juslinės analizės laboratorijos atstovą.*

Aš, ....................................................................................., atsižvelgiant į aukščiau išdėstytas sąlygas ir aplinkybes **sutinku** dalyvauti KTU Maisto instituto, juslinės analizės laboratorijos atliekamame tyrime ***„Augalinės kilmės mėsos analogų, su ekstruduotomis sojos išspaudomis, juslinė analizė“***

Tyrimo dalyvis ....................................................................................................................................................

(parašas, vardas, pavardė, data)

**CONSENT TO PARTICIPATE IN THE RESEARCH**

KTU Institute of Food, Laboratory of Sensory Analysis, is conducting a research study:

***„Sensory evaluation of plant-based meat analogues with extruded soy press-cakes“***

**Aim of the study.** To carry out a descriptive quantitative sensory analysis of a plant-based product enriched with extruded soya bean press-cakes.

**Participants in the study.** The evaluators are experts experienced in descriptive sensory analysis.

**Method of the study.** The study will be conducted in accordance with the requirements of ISO 11035:1994 (Sensory analysis - Identification and selection of descriptors for establishing a sensory profile by a multidimensional approach). During the study, the evaluator will be presented with different samples of meat analogues and asked to answer questions while tasting the products. The evaluation samples and the evaluation questionnaire will be provided to each evaluator personally, providing a private space for the evaluation. The evaluation is anonymous and it will not be possible to identify which evaluator has provided the answers from the evaluation results. The duration of the evaluation is approximately 25 minutes.

**Allergenicity warning: the product contains soya.**

*Participant's rights: The participant is guaranteed anonymity and confidentiality of the data obtained. The aggregated results of the study will only be used for the purpose of the study. The participant may withdraw from the study before the start of the study, but once the evaluation has been submitted, there is no possibility to withdraw it, as the evaluation is completely anonymous and it is not possible to identify which evaluation belongs to a particular evaluator. Evaluators under the age of 18 must provide a signed consent form from at least one of their parents/guardians in order to take part in the study, and must express their agreement to participate in the study. In case of any questions, the participant may contact a representative of the KTU Food Institute, Sensory Analysis Laboratory by e-mail Aelita.Zabulione@ktu.lt.*

I, ....................................................................................., subject to the above conditions and circumstances, agree to participate in the study **"Sensory analysis of plant-based meat analogues with extruded soybean press-cakes"** conducted by the KTU Institute of Food, Laboratory of Sensory Analysis.

Evaluatos ....................................................................................................................................................

(name, surname, signature, date
