# Supplementary material for: Effects of extrusion conditions on the morphological, functional, and sensory properties of soy press cake extrudates
Source: Heliyon. 2024 Jun 19;10(12):e32614. doi: 10.1016/j.heliyon.2024.e32614 (PMC11252654; doi:10.1016/j.heliyon.2024.e32614)
Supplement: Multimedia component 2 [file mmc2.docx]

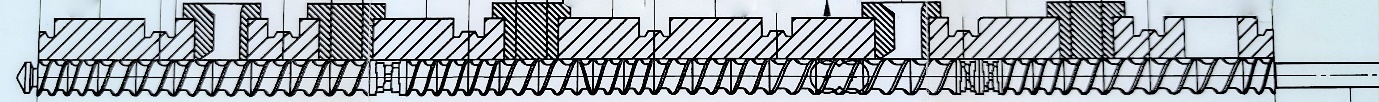


C_1_

C_2_

C_3_

C_1_

K_1_

C_2_

C_3_

C_1_

C_R_

C_3_

C_1_

K_2_

C_3_

C_1_

**Supplementary Figure 1.** Configuration of the extruder screws: *C_1_* – conveying screw element with a length of 25 mm and pitch of 12.5 mm, *C_2_* – conveying screw element with a length of 37.5 mm and pitch of 18.75 mm, *C_3_* – conveying screw element with a length of 31.25 mm and pitch of 15.625 mm, *C_R_* – reverse conveying screw element with a length of 12.5 mm and pitch of 6.25 mm, *K_1_* – kneading screw element with a length of 18.75 mm, *K_2_* – kneading screw element with a length of 31.25 mm
