# Supplementary material for: Effects of extrusion conditions on the morphological, functional, and sensory properties of soy press cake extrudates
Source: Heliyon. 2024 Jun 19;10(12):e32614. doi: 10.1016/j.heliyon.2024.e32614 (PMC11252654; doi:10.1016/j.heliyon.2024.e32614)
Supplement: Multimedia component 3 [file mmc3.docx]

Vertintojas: ____________________________

Data:___________________________

Mėginio kodas:____________________

Atkreipkite dėmesį, kad 1 = savybė visiškai nejaučiama, 12 = jaučiama maksimaliai. „X“ pažymėkite savybės intensyvumą.

|  | 1 | 2 | 3 | 4 | 5 | 6 | 7 | 8 | 9 | 10 | 11 | 12 |
| --- | --- | --- | --- | --- | --- | --- | --- | --- | --- | --- | --- | --- |
| Bendras kvapo intensyvumas |  |  |  |  |  |  |  |  |  |  |  |  |
| Popieriaus kvapas |  |  |  |  |  |  |  |  |  |  |  |  |
| Saldus kvapas |  |  |  |  |  |  |  |  |  |  |  |  |
| Rūgštus kvapas |  |  |  |  |  |  |  |  |  |  |  |  |
| Augalinės žaliavos kvapas |  |  |  |  |  |  |  |  |  |  |  |  |
| Pašalinis kvapas |  |  |  |  |  |  |  |  |  |  |  |  |
|  | | | | | | | | | | | | |
| Masės standumas |  |  |  |  |  |  |  |  |  |  |  |  |
| Skaidulų standumas |  |  |  |  |  |  |  |  |  |  |  |  |
| Sultingumas |  |  |  |  |  |  |  |  |  |  |  |  |
| Riebalingumas |  |  |  |  |  |  |  |  |  |  |  |  |
| Burnos padengimas |  |  |  |  |  |  |  |  |  |  |  |  |
|  | | | | | | | | | | | | |
| Bendras skonio intensyvumas |  |  |  |  |  |  |  |  |  |  |  |  |
| Saldumas |  |  |  |  |  |  |  |  |  |  |  |  |
| Sūrumas |  |  |  |  |  |  |  |  |  |  |  |  |
| Rūgštumas |  |  |  |  |  |  |  |  |  |  |  |  |
| Kartumas |  |  |  |  |  |  |  |  |  |  |  |  |
| Aitrumas |  |  |  |  |  |  |  |  |  |  |  |  |
|  | | | | | | | | | | | | |
| Riešutų skonis |  |  |  |  |  |  |  |  |  |  |  |  |
| Kreidos skonis |  |  |  |  |  |  |  |  |  |  |  |  |
| Popieriaus skonis |  |  |  |  |  |  |  |  |  |  |  |  |
| Mėsiškas skonis |  |  |  |  |  |  |  |  |  |  |  |  |
| Pašalinis skonis |  |  |  |  |  |  |  |  |  |  |  |  |
| Poskonio intensyvumas |  |  |  |  |  |  |  |  |  |  |  |  |

Evaluator: ____________________________

Date:___________________________

Sample code:____________________

Please note, that 1 = can not feel at all, 12 = maximal possible sensation. Please mark „X“ along with intensity that you feel.

|  | 1 | 2 | 3 | 4 | 5 | 6 | 7 | 8 | 9 | 10 | 11 | 12 |
| --- | --- | --- | --- | --- | --- | --- | --- | --- | --- | --- | --- | --- |
| Overall odour intensity |  |  |  |  |  |  |  |  |  |  |  |  |
| Cardboard odor |  |  |  |  |  |  |  |  |  |  |  |  |
| Sweet odor |  |  |  |  |  |  |  |  |  |  |  |  |
| Sour odor |  |  |  |  |  |  |  |  |  |  |  |  |
| Plant origin odor |  |  |  |  |  |  |  |  |  |  |  |  |
| Off-odour |  |  |  |  |  |  |  |  |  |  |  |  |
|  | | | | | | | | | | | | |
| Firmness |  |  |  |  |  |  |  |  |  |  |  |  |
| Fibre firmness |  |  |  |  |  |  |  |  |  |  |  |  |
| Juicyness |  |  |  |  |  |  |  |  |  |  |  |  |
| Greasiness |  |  |  |  |  |  |  |  |  |  |  |  |
| Mouthcoating |  |  |  |  |  |  |  |  |  |  |  |  |
|  | | | | | | | | | | | | |
| Overall taste intensity |  |  |  |  |  |  |  |  |  |  |  |  |
| Sweet taste |  |  |  |  |  |  |  |  |  |  |  |  |
| Salty taste |  |  |  |  |  |  |  |  |  |  |  |  |
| Sour taste |  |  |  |  |  |  |  |  |  |  |  |  |
| Bitter taste |  |  |  |  |  |  |  |  |  |  |  |  |
| Astringent taste |  |  |  |  |  |  |  |  |  |  |  |  |
|  | | | | | | | | | | | | |
| Nutty taste |  |  |  |  |  |  |  |  |  |  |  |  |
| Chalky taste |  |  |  |  |  |  |  |  |  |  |  |  |
| Cardboard taste |  |  |  |  |  |  |  |  |  |  |  |  |
| Meaty taste |  |  |  |  |  |  |  |  |  |  |  |  |
| Off-taste |  |  |  |  |  |  |  |  |  |  |  |  |
| Aftertaste intensity |  |  |  |  |  |  |  |  |  |  |  |  |
